# Folic Acid: Sources, Chemistry, Absorption, Metabolism, Beneficial Effects on Poultry Performance and Health

**DOI:** 10.1155/2022/2163756

**Published:** 2022-08-19

**Authors:** Herinda Pertiwi, Mohamad Yusril Nur Mahendra, Juriah Kamaludeen

**Affiliations:** ^1^Department of Health Studies, Faculty of Vocational Studies, Airlangga University, Jalan Dharmawangsa Dalam 28-30, Surabaya 60286, Indonesia; ^**2**^ Department of Animal Science and Fishery, University Putra Malaysia Bintulu Serawak Campus, Nyabau Road, Bintulu 97008, Serawak, Malaysia

## Abstract

Recently, there has been an increasing interest in the study of the effects of folic acid (FA) on poultry because it was observed that FA could overcome problems in poultry health while improving its performance. FA, or folate, is a water-soluble B vitamin essential in poultry, so FA intake must be available in the feed. Sources of FA in feed come from plants or animals, and animal sources have relatively more stable FA. The ingested FA will be absorbed in the intestinal lumen and transported into the liver through the blood vessels. Therefore, FA has a positive effect on the performance and health status of poultry. The effect of FA on poultry performance is to increase reproductive tract development, FA content in eggs, hatchability, weight gain, average initial body weight, feed intake, relative growth rate, chick body weight, breast fillet percentage, and reduce FCR and white striping score. At the same time, the effect on poultry health influences antioxidant activities, thyroid hormones, blood biochemicals, anti-inflammatory gene expressions, and immune responses. The present review deals with FA sources, chemistry, absorption, metabolism, effects on performance, and poultry health, which are based on valid basic information.

## 1. Introduction

Global human population growth has increased the demand for food, including animal protein, primarily satisfied by poultry products [1]. Therefore, poultry farming is developing rapidly, especially in developing countries [2]. However, along with the development of poultry farming, several problems, such as white striping and fat accumulation, adversely affect poultry carcass quality and feed efficiency [3,4], which is more common in chickens with a fast-growing genotype such as Cobb than in slow-growing chickens [5]. This decrease in quality will initiate economic losses for farmers [6]. Factors that stimulate these problems include genetics, environment, and nutrition [7].

Research on the effects of nutrients such as folic acid (FA) has been carried out to treat several problems in poultry. Yu et al. [8] reported that FA supplementation could reduce lipid accumulation per cell in chickens. Moreover, lipids in chicken hepatocytes and in abdominal fat deposits can also be reduced by FA supplementation [9,10]. It could also influence glucose and lipid metabolism in broiler chickens [11].

Folic acid, also known as folate, is a water-soluble B vitamin with a pteroylglutamic acid backbone [12]. Exogenous intake of FA is necessary because FA is an essential vitamin that acts as a cofactor and reaction cosubstrate in synthesizing amino and nucleic acids that are useful in nucleic acid synthesis, physiological processes, and regeneration of methionine [13]. Meanwhile, lacking folic acid supplementation could disrupt animal physiological functions, growth inhibition, and biochemistry [14]. For example, a lack of FA intake in pigs may change the expression of specific genes, the amount of longissimus dorsi muscle fibers, and intramuscular triglyceride levels [15].

Folic acid supplementation is easily absorbed by the animal's body, such as in eggs, thus lowering the cost of fulfilling animal products' nutrition [16]. Moreover, supplementing the poultry diet with FA could increase the consumption of FA in the human diet to reduce neural tube defects in infants [17]. El-Husseiny et al. [14] show that folic acid could affect the value of relative economic efficiency, especially in developing countries. In addition, research conducted by Al-Saffar and Rose [18] showed that optimal amino acid supplementation in feed could positively affect economic efficiency. The relative economic efficiency (REE) and the highest economic efficiency (EE) were also positively affected by folic acid supplementation [19].

Previous studies have also concluded that folic acid positively affects various animals. For example, Kumar et al. [20] observed that reduction of FA in the diet of female rats could improve lipid and visceral fat metabolism in their offspring. Meanwhile, FA deficiency in rats could also initiate memory impairment in mice [21]. However, a review of the effects of FA on poultry has not been widely carried out. Therefore, this review compares the effects of folic acid supplementation on poultry's production performance and health and discusses sources, chemistry, absorption, and metabolism.

## 2. Sources and Chemistry of Folic Acid

Animals obtain folic acid intake from vegetables, grasses, green leaves, mushrooms, yeast, and citrus. In contrast, animal sources of FA are liver and eggs, where FA from animal sources is more stable to heat changes [22].

**Figure 1:**
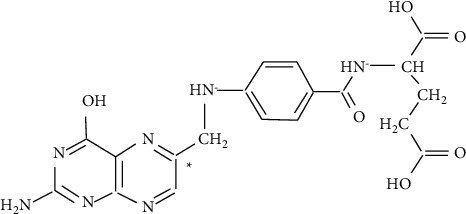
Structure of folic acid.

Folic acid has the molecular formula C_19_H_19_N_7_O_6_, a molecular weight of 441.4, a melting point of 250°C, a density of 1.68 g/cm^3^, and is susceptible to the temperatures used in feed processing such as expansion, conditioning, and extrusion [23]. The IUPAC name of FA is (*2*S)-2-((4-(((2-amino-4-oxo-1,4-dihydropteridin-6-yl)methyl)amino)benzoyl)amino)pentanedioic acid, while several synonyms for FA such as vitamin B_9_, vitamin M, folacin, pteroyl-L-glutamic acid, 2-amino-6-((p-((1,3-dicarboxypropyl)carbamoyl)aniline)methyl)-4-pteridinol; *N*-(4-(((2-amino 1,4-dihydro-4-oxo-6-pteridinyl)methyl)amino)benzoyl)-L-glutamic acid), while the Chemical abstractsservice (CAS) number is 59-30-3 [22]. The structure of folic acid can be seen in (Figure 1).

The synthesis of FA occurs in 4 stages, beginning with the formation of N-4-nitrobenzoyl-L-glutamic acid through the reaction between 4-nitrobenzoyl chloride with monosodium L-glutamate, then forming N-4-aminobenzoyl-L-glutamic acid with catalytic hydrogenation, condensed using 2,4,5-triamino-6-hydroxypyrimidine and 1,1,3-trichloroacetone, and finally multi-processed to purify the product [22].

Folic acid could show a solubility of 561 mg/L at pH 6 when crystallized four times. FA also had varying fractions (5–80%) of particle level, with a diameter of <50 m and a dust potential of 0.5 g/m^3^ [24]. Moreover, FA in the form of yellowish crystalline powder is odorless, tasteless, dry, and relatively stable to moisture and heat. Still, it becomes unstable when exposed to light and pH below 5.0 [25].

## 3. Folic Acid Absorbtion and Metabolism

The recommended level of FA by the national research council (NRC) is 0.55 mg/kg of feed. Then further research has determined that FA levels should be 3 mg/kg [26]. Meanwhile, according to Bagheri et al. [27], the recommended value of FA in laying hens is 0.25 mg/kg could produce a significant product. The NRC recommended levels of folic acid in different poultry diets can be seen in (Table 1).

Plant folate is mainly in the form of polyglutamate with 5–9 glutamate tails [28]. By folate hydrolase, polyglutamate is converted to monoglutamate [29]. Meanwhile, FA transport system-related enzymes are expressed by the chicken intestinal tract's four-segment intestinal tract transporter [30].

FA could increase its absorption under pH 6.0 conditions in the chicken digestive tract, with an increase in the jejunum and duodenum while it decreases in the ceca and ileum [31]. Moreover, the absorption rate of FA in the chicken jejunum from the mucosa to the serosa could reach a plateau when the concentration is more than 0.1 M [32]. After being absorbed in the small intestine, folic acid is transported by the portal vein to the liver [33].

When in the liver, FA will be metabolized by several enzymes such as dihydrofolate reductase (DHFR), 5,10-methylenetetrahydrofolate reductase (5,10-MTHFR), and tetrahydrofolic acid (THF) [7]. FA catalyzes the reduction of dietary FA and dihydrofolate in the liver to become THF, while the central reaction in folate metabolism is carried out by 5,10-MTHFR [34]. Meanwhile, synthesizing amino acids, nucleic acids, and tetrahydrofolic acid participates as a one-carbon unit donor and receptor [34,35]. Furthermore, FA and (6s)-5,6,7,8-tetrahydrofolate substrates could inhibit DHFR-mediated dihydrofolate reduction [36,37]. In the research of Bai et al. [30], 10 mg/kg FA supplementation decreased duodenal DHFR. Furthermore, when folate transport becomes saturated in erythrocytes, a certain amount of FA is excreted through the feces [38]. Proton-coupled folate transporter (Pcft) jejunal mRNA expression, reduced folate carrier (Rfc), duodenal folate receptor (Folr), DHFR, Abcc2, Abcc3, and Abcc5 were used to determine the level of FA absorption in the chicken digestive tract, where the process of entry of folate into the vasculature of erythrocytes is related to the expression of Abcc3 and Abcc5.

**Figure 2:**
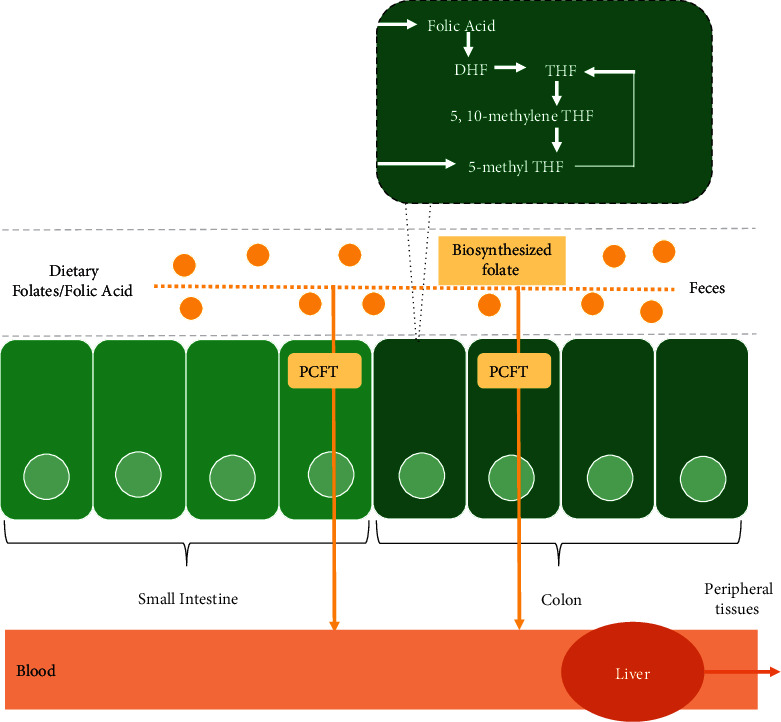
Folic acid absorption and metabolism.

In contrast, the admission of folate into the intestinal lumen is associated with the expression of Abcc2 [31]. In a study by Jing et al. [39], 10 mg/kg FA supplementation decreased jejunal Rfc expression. Excessive FA supplementation could negatively affect the expression of Pcft and Rfc in protein levels [40]. FA metabolism is a complex process; in short, it occurs with the transport of FA from the intestinal lumen to erythrocytes, then into the bloodstream to enter the liver. Illustrations of FA absorption and metabolism can be seen in (Figure 2).

## 4. Effect of Folic Acid on Animal Production

Folic acid needs must be met to support animal growth and reproduction, which could negatively impact normal animal physiological functions (Table 2) [12]. The FA requirement in poultry is influenced by many factors such as strain, age, production stage, primary feed, FA level, environmental factors, feed, and management protocol [41]. Saturation of plasma folate concentration could be achieved after chickens are supplemented with folic acid by as much as 2–4 mg/kg [8]. Furthermore, the research of Bai et al. [30] showed that on FA, as much as 6 mg/kg was sufficient to reach the plateau. In broiler chickens, female chickens require a higher intake of folic acid than males [42]. In laying hens, FA is needed to develop the oviducts, whereas in hens, a lack of vitamins will result in defective albumen deposition [43].

Krishnan [44] reported that 2 and 4 ppm FA supplementation for eight weeks increased egg production. Moreover, Terčič and Pestotnik [41] found that feeding laying hens a 50 mg/kg FA diet for four weeks might increase the amount of FA in eggs while also improving hatchability (percent) and chick body weight by one percent, which might be explained by the FA group's lower water diffusion via the shell pores. The study by Dickson et al. [45] revealed an increase in the FA content of eggs as much as 46.9–57.9 g/eggs with FA supplementation of 4 mg/kg for 11 periods in 28 days. Bai et al. [30] found that FA supplementation in laying hens by as much as 6 mg/kg for eight weeks could increase egg yolk folate content. In turkeys, FA supplementation of as much as 1–2 mg/kg could increase the vitamin content in eggs, chick weight, and growth [43]. However, Hebert et al. [46] showed that supplementing 4 mg/kg of crystalline FA for 21 days could increase folate deposition in eggs. However, another study concluded that adding FA as much as 4.0 and 8.0 mg/kg to quail feed for six weeks did not affect egg yolk color, egg haugh, egg yolk, and albumen pH [21]. Meanwhile, Tactacan et al. [31] also concluded that FA supplementation of 10 or 100 mg/kg for two weeks did not affect the egg weight of Shaver White.

The research of Bagheri et al. [27] showed that 5–15 mg/kg FA supplementation in layers for six weeks significantly reduced FCR, increased egg mass and weight, and increased 5-MTHF content in egg yolks. In one-carbon unit transfer, 5-MTHF acts as a catalytic substrate [47]. Meanwhile, in the research of Gouda et al. [25], FA supplementation of 1.5 mg/kg for 35 days increased weight gain, average initial body weight, feed intake, and relative growth rate. According to Terčič and Pestotnik [41], the average daily consumption of poultry was higher when 32 mg/kg FA was added to the feed.

In terms of meat quality, FA could reduce white striping scores and increase the percentage of regular breast fillets [42]. Decreased white stripe scores were associated with lipid metabolism. Liu et al. [9] reported that FA 15 mg/L could affect lipid metabolism by interfering with fatty acid synthesis and promoting triglyceride hydrolysis. Meanwhile, increased fat deposits could initiate white-striped breast muscle fillets [48]. Folate-deficient animals will increase lipid droplets in their offspring [28]. However, the research of Zhang et al. [12] showed that FA supplementation of 2.0 mg/kg for 12 weeks had no significant effect on abdominal fat related to diet, age, and species.

The combination of 13 mg/kg of FAwith 0.25% meth and B12 for 11 weeks could also increase egg weight, hatchability, feed conversion ratio, and chicken weight, while blood parameters were insignificant [14]. Ezzat et al. [49] reported that dietary supplementation of the Matrouh poultry strain with Betaine (BET) (1.0 g/kg) and FA (1.0 mg/kg) at 24–36 weeks in the Egyptian summer increased shell thickness, Haugh units, shell to egg weight, sperm motility, and decreased dead spermatozoa.

Folic acid must be present in the feed to improve oviduct development, egg FA content, hatchability, weight gain, chick body weight, initial average body weight, feed intake, relative growth rate, appropriate breast fillet percentage, and decrease FCR and white striping score.

## 5. Effect of Folic Acid on Animal Health

Folic acid intake is needed to maintain animal health, growth, and development [13]. However, the response of layer chickens to FA supplementation varies depending on age. In older chickens, the response is not as good as in young chickens [50]. Meanwhile, a lack of FA intake could initiate foot problems in poultry and initiate eye disease, anemia, and cancer [26,51–53]. Furthermore, there was no toxic effect of FA on chickens [54]. Folic acid is also involved in metabolic processes by influencing serine, glycine, histidine, methionine, choline, and thiamine; it also plays a role in the transfer of monocarbonate and affects the synthesis of purines and pyrimidines so that it plays a role in the formation of nucleic acids needed for cell division [43].

Gouda et al. [25] found that feed supplementation with FA at a level of 1.5 mg/kg for 35 days could improve broiler antioxidant status by increasing heat shock protein 70 (HSP70), total antioxidant capacity (TAC), catalase enzyme (CAT), and superoxide dismutase enzyme (SOD) activity under heat stress, as well as the levels of thyroid hormones, triiodothyronine (T3), and thyroxin (T (NDV). Ezzat et al. [49] also reported that supplementation with betaine (1 g/kg diet), vitamin C (200 mg/kg diet), and FA (1 mg/kg diet) alone or in combination for 12 weeks significantly increased antibody titer against SRBC'S, hemoglobin, globulin, and total protein, and significantly decreased serum glucose, cholesterol, HDL, and triglycerides. In comparison, a diet of 4 mg of FA/kg for eight weeks could impact old laying hens by lowering serum uric acid and young laying hens by lowering serum glucose levels [50].

The supplementation of FA by as much as 5–10 mg/kg in the diet could also reduce the droplet size of liver lipids and LPL, PPAR*γ*, and FAS in abdominal fat, indicating that FA affects FA gene expression in the liver [7]. Moreover, Yu et al. [8] found that the supplementation of folate 0–16 mg/L could reduce FAS and CCAAT/enhancer-binding protein (C/EBP*α*) gene expression by altering the methylation level of the gene promoter. Liu et al. [9] found that FA by 15 mg/L in the culture medium could suppress de novo fatty acid synthesis and coordinately promote triglyceride hydrolysis and export in primary chicken hepatocytes from newborn chicks. In contrast, total cholesterol, HDL, HDL-c, LDL, LDL-c, apolipoprotein A, and apolipoprotein B had no significant effect on FA supplementation, while VLDL levels increased. Another study revealed that folate deficiency could increase lipid droplets and LPL and IGF2 gene expression in the liver of offspring [28].

Supplementing FA by 4 mg/kg in the young layer for eight weeks will increase the biochemical constituent, total IgG. It could initiate a pleiotropic effect in the inflammatory response, and it was observed that the expression of IL-1*β* and IL-18 was downregulated after exposure to an acute LPS challenge. Still, its expression in the spleen has increased [55]. Meanwhile, Jing et al. [50] concluded that FA supplementation of 4 mg/kg for eight weeks in laying hens could reduce the inflammatory response after LPS induction by inhibiting mitogen-activated protein kinase and the nuclear factor-kappa B pathways in RAW 264.7 cells. According to Kennedy [13], the anti-inflammatory effect of FA occurs by reducing the circulation of inflammatory mediators.

**Figure 3:**
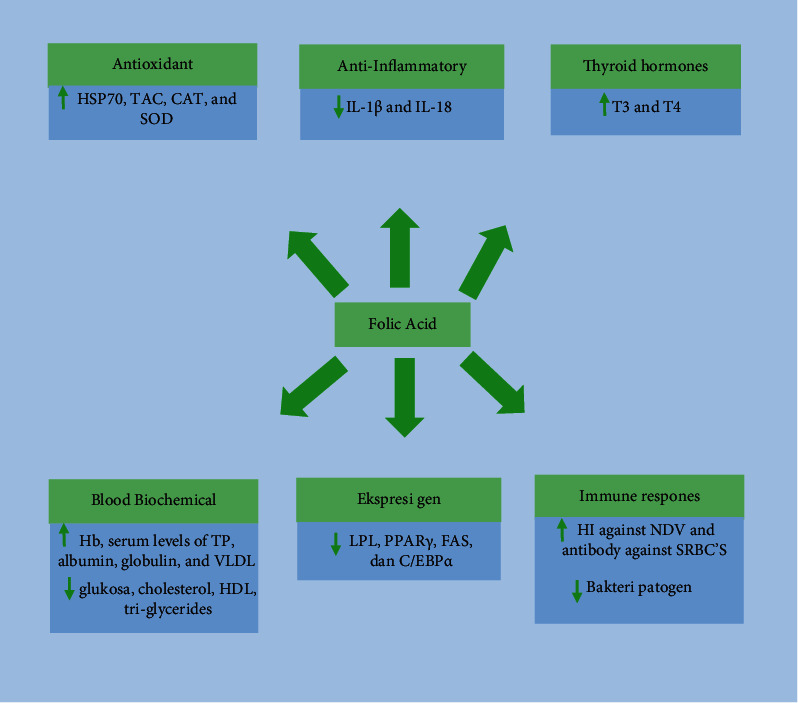
Effect of folic acid on animal health.

Folic acid could also affect cecal microbes in laying hens. High FA consumption resulted in a lower relative abundance of *Fusobacteria*, *Saccharibacteria*, *Eremiobacterota,* and *Verrucomicrobia*. In contrast, bacterial pathogens such as *Bifidobacteriaceae* decreased after supplementation with 24 mg/kg FA for eight weeks [30]. Excess FA will be transferred to the cecum, where the cecum contains many beneficial microbes to maintain chicken health [56,57]. Some microbes found in the cecum can also synthesize folates, such as *Fusobacteria* and *Proteobacteria* [58]. In short, FA is needed by poultry to maintain nutritional status, which has activity on antioxidants, thyroid hormones, blood biochemicals, anti-inflammatory, gene expression, and immune response, which could be seen in (Figure 3). Briefly, the effect of folic acid on poultry health can be seen in (Table 3).

## 6. Conclusion

In this review, we have provided specific information regarding the origin, chemistry, absorption, and metabolism of folic acid, which demonstrates its ability to boost productivity by increasing weight gain, average beginning body weight, feed intake, egg weight, and nutrient content. Also, it improves the health status of poultry by exhibiting antioxidant, anti-inflammatory, and immune response activities against pathogens and lowering cholesterol levels. Therefore, this study recommends regular and appropriate FA acid supplementation to provide optimal results. However, further studies are needed to open up new dimensions regarding the utilization of folic acid in a broader range of poultry species.

## Figures and Tables

**Table 1:** NRC recommended levels of folic acid in different poultry diets.

Type of poultry	Age	Folic acid requirement (mg/kg)
White leghorn	0–6 Weeks	0.55
	6–12 Weeks	0.25
	12–18 Weeks	0.25
	18 Weeks to the first egg	0.25

Brown leghorn	0–6 Weeks	0.52
	6–12 Weeks	0.23
	12–18 Weeks	0.23
	18 Weeks to the first egg	0.23

Broiler strain	0–28 Days	0.30
New Hampshire	0.33–1.45 Days	0–35
Rhode Island red	1–21 Days	≤0.3
White Plymouth	1–28 Days	≤0.3
Arbor Acres	1–20 Days	0.3–0.45
Bronze	0–6 Days	0.8
Females bronze	32–48 Days	0.7
Jersey buff	0–3 Days	2.0
Females large white	32–48 Days	1.23

Turkeys males/females	0–4 Weeks	1.0
	4–8 Weeks	1.0
	8–12/8–11 Weeks	0.8
	12–16/11–14 Weeks	0.8
	16–20/14–17 Weeks	0.7
	20–24/17–20 Weeks	0.7

**Table 2:** Effect of folic acid in the animal.

Animal	Dose rate	Major findings	References
Laying hens	Basal diet added FA 4 mg/kg	Increased egg weight and egg mass, decreased serum glucose levels, and serum uric acid	Jing et al. [50]

Laying hens	Basal diet added FA 10 or 100 mg/kg	Increase egg and plasma folate concentrations and also decrease the transport of FA in the duodenum	Tactacan et al. [54]

Laying hens	Basal diet added 10 mg/kg of FA	Affects the activity of plasma homocysteine, methionine synthase, and hepatic serine hydroxymethyltransferase. And egg folate concentrations	Tactacan et al. [59]

Laying hens	FA added 4 mg/kg	Enriches the FA content of eggs	Dickson et al. [45]

Laying hens	Basal diet added with chromium yeast (150 mg ton^−1^) + FA (10 mg ton^−1^)	Increase in the feed conversion, decrease in feed consumption, and egg yolk cholesterol	Eseceli et al. [60]

Laying hens	Basal diet added FA 50 mg/kg	Increase the body weight of the newly hatched chicks	Terčič and pestotnik [41]

Laying hens	Basal diet added FA 24 mg/kg	It affects the cecum by reducing pathogenic bacteria	Bai et al. [30]

Laying hens	Basal diet added FA 5, 10, and 15 mg/kg	It affects egg yolks by increasing 5-MTHF content and egg production	Bagheri et al. [27]

Laying hens	Basal diet added 4 mg/kg of crystalline FA	Affect the accumulation of folate in eggs	Hebert et al. [45]

Laying hens	Basal diet added 2 and 4 ppm FA	Increased egg production	Krishnan [43]

Laying hens	Basal diet added BET (1,0 g/kg) and FA (1,0 mg/kg)	Increases in haugh units, egg weight, shell thickness, sperm motility, and decreased dead spermatozoa	Ezzat et al. [49]

Hepatocytes from new-hatched male chicks	The concentration of FA in the culture medium is 15 mg/L	Inhibits de novo fatty acid synthesis and promotes hydrolysis and exportation of triglyceride in primary chicken hepatocytes	Liu et al. [9]

Broilers	Basal diet added 200 mg L-ascorbic acid plus 1.5 mg FA/kg	Can improve the antioxidant status, growth, and health status of broilers under heat stress	Gouda et al. [25]

Broilers	2.0 mg/kg folate added to feed	Less significantly affect product performance and slaughter performance	Zhang et al. [12]

Broilers	FA (13.0 mg/kg), meth (0.25%), and B12 (0.15 mg/kg) were added to feed	Positive effect on FCR, egg weight, hatchability, and weight of DOC	El-Husseiny et al. [48]

Broilers	FA was added to the feed at 5 mg/kg and 10 mg/kg	Reduce fat accumulation by regulating gene expression	Zhang et al. [7]

Broilers	The basal diet was supplemented with 0.25, 1.25, 2.50, and 5.00 mg/kg^−1^ folate	Could regulate glucose and lipid metabolism in broilers	Wu et al. [11]

Laying quails	FA as much as 4 and 8 mg/kg added to the feed	Significantly increase FA content and egg yolk color	Sadegheymojarad et al. [21]

Turkey	Basal diet added FA 1–2 mg/kg	Increase the vitamin content in eggs, increase the weight of chicks, and their growth	Barroeta et al. [43]

**Table 3:** Effect of folic acid on poultry health.

Animal	Dose rate	Major findings	References
Broiler	Basal diet added L-ascorbic acid (AA) 200 mg/kg and FA 1.5 mg/kg	Increase thyroid hormone, hemoglobin, insulin growth factor, albumin, globulin, total protein, antioxidant activity, and antibodies against viruses	Gouda et al. [25]

Laying hens	Basal diet added FA 24 mg/kg	Reducing pathogens in the cecum	Bai et al. [30]

Broiler	Basal diet added ten ppm FA	Increases bile acid concentration and heart weight	Fisayo et al. [61]

Laying hens	Basal diet added FA 4 mg/kg	Lowers serum glucose and uric acid levels	Jing et al. [50]

Laying hens	Basal diet added FA 4 mg/kg	Enhances biochemical constituents, IgG, and exhibits pleiotropic activity	Munyaka et al. [55]

Broiler	15 mg/L in the culture of primary chicken hepatocytes	Inhibits de novo fatty acid synthesis and can promote triglyceride hydrolysis	Liu et al. [9]

Broiler	Poultry supplemented with FA 800 mg/l	Can reduce liver lipogenesis, suppress adipocyte proliferation and differentiation	Liu et al. [10]

Broiler	Poultry supplemented with FA 16 mg/L	Increase adipocyte proliferation	Yu et al. [8]

Broiler	Basal diet added FA 0.25–5.00 mg/kg	Affect lipid and glucose metabolism in chicken progeny	Wu et al. [11]

Broiler fertile eggs	50–150 *μ*g folic acid was injected into eggs	Upregulates IGF2 expression and aids organ development	Liu et al. [62]

## Data Availability

Information about folic acid was retrieved from a literature search of electronic databases such as PubMed, Elsevier, Research Gate, Academia, and Google Scholar. The keywords used to perform the search were: folic acid in poultry, antioxidant, folic acid, and folic acid health protective. The research data are presented in tables, diagrams, and graphs in the articles. Supportive data for discussion and comparison were from previous studies which have been cited from recent journal related to the focus of this article. These data are publicly available and accessible online. Detailed sources are provided in References of the manuscript
